# Superior performance of biocomposite nanoparticles PLGA-RES in protecting oocytes against vitrification stimuli

**DOI:** 10.3389/fbioe.2024.1376205

**Published:** 2024-03-11

**Authors:** Guiping Hai, Jiachen Bai, Yucheng Liu, Jun Li, Aiju Liu, Jingjing Wang, Qian Liu, Weijun Liu, Pengcheng Wan, Xiangwei Fu

**Affiliations:** ^1^ College of Animal Science, Xinjiang Agricultural University, Ürümqi, China; ^2^ State Key Laboratory of Sheep Genetic Improvement and Healthy Breeding, Institute of Animal Husbandry and Veterinary Sciences, Xinjiang Academy of Agricultural and Reclamation Sciences, Shihezi, China; ^3^ Department of Reproductive Medicine, Reproductive Medical Center, The First Hospital of Hebei Medical University, Shijiazhuang, China; ^4^ National Engineering Laboratory for Animal Breeding, Key Laboratory of Animal Genetics, Breeding and Reproduction of the MARA, Beijing Key Laboratory for Animal Genetic Improvement, State Key Laboratory of Animal Biotech Breeding, College of Animal Science and Technology, China Agricultural University, Beijing, China

**Keywords:** nanoparticles, PLGA-RES, oocytes, endocytosis, vitrification

## Abstract

Irreversible cryogenic damage caused by oocyte vitrification limits its widespread use in female fertility preservation. In recent years, nanoparticles (NPs) have gained great attention as potential alternatives in protecting oocytes against cryoinjuries. In this paper, a novel composite nanoparticle, poly (lactic-co-glycolic acid)-resveratrol (PLGA-RES) was designed to improve the biocompatibility and sustained release properties by encapsulating natural antioxidant RES into PLGA NPs. Firstly, biotoxicity and oxidation resistance of PLGA-RES were determined, and the results showed that PLGA-RES had nontoxic effect on oocyte survival during *in vitro* maturation (IVM) (97.08% ± 0.24% vs. 98.89% ± 1.11%, *p* > 0.05). Notably, PLGA-RES even increased maturation (65.10% ± 4.11% vs. 52.85% ± 2.87%, *p* < 0.05) and blastocyst rate (56.13% ± 1.36% vs. 40.91% ± 5.85%, *p* < 0.05). Moreover, the reduced reactive oxygen species (ROS) level (13.49 ± 2.30 vs. 34.07 ± 3.30, *p* < 0.01), increased glutathione (GSH) (44.13 ± 1.57 vs. 37.62 ± 1.79, *p* < 0.01) and elevated mitochondrial membrane potential (MMP) levels (43.10 ± 1.81 vs. 28.52 ± 1.25, *p* < 0.01) were observed in oocytes treated with PLGA-RES when compared with that of the control group. Subsequently, the role of PLGA-RES played in oocytes during vitrification was systematically evaluated. The results showed that the addition of PLGA-RES during vitrification and thawing significantly improved the survival rate (80.42% ± 1.97% vs. 75.37% ± 1.3%, *p* < 0.05). Meanwhile, increased GSH (15.09 ± 0.86 vs. 14.51 ± 0.78, *p* < 0.01) and mitochondrial membrane potential (22.56 ± 3.15 vs. 6.79 ± 0.60, *p* < 0.01), decreased reactive oxygen species levels (52.11 ± 2.95 vs. 75.41 ± 7.23, *p* < 0.05) and reduced mitochondrial abnormality distribution rate (25.00% ± 0.29% vs. 33.33% ± 1.15%, *p* < 0.01) were assessed in vitrified MII oocytes treated with PLGA-RES. Furthermore, transcriptomic analyses demonstrated that PLGA-RES participated in endocytosis and PI3K/AKT/mTOR pathway regulation, which was verified by the rescued expression of ARRB2 and ULK3 protein after PLGA-RES treatment. In conclusion, PLGA-RES exhibited potent antioxidant activity, and could be used as an efficacious strategy to improve the quality of vitrified oocytes.

## 1 Introduction

Nanobiotechnology has been used in a wide range of biomedical applications, including bio-detection, drug delivery and diagnostic imaging, particularly in the area of cancer diagnosis and treatment (Wang et al., 2021). Even some popular nanobiotechnology, such as electrospinning (Qian et al., 2023; Wang et al., 2023; Duan et al., 2024) and electrospray (Sun et al., 2023; Zhou et al., 2023), make it easier and more efficient to produce nanofiber/microparticle for disease treatment. In the last decades, many synthetic polymers have been investigated for nanomedical applications, especially as drug delivery systems. For this purpose, polymers must be nontoxic, biodegradable and biocompatible (Narmani et al., 2023). PLGA is a degradable aliphatic polyester, which has been approved for clinical use by the Food and Drug Administration (FDA) (Sousa de Almeida et al., 2021). Notably, PLGA was considered to be effective carriers for drug delivery due to its good biocompatibility, excellent safety profile and tunable rate of biodegradation *in vivo* (Benhabbour et al., 2019; Rocha et al., 2022; Shakya et al., 2023). Although the ability of such polymers to deliver various therapeutic agents in a targeted and/or sustained release manner has been extensively evaluated (Dristant et al., 2023), the potential role of PLGA loaded drug delivery in oocytes exposed to external stimuli remains elusive.

Oocyte cryopreservation contributes greatly to fertility preservation and endangered species conservation, however, oxidative stress-induced cryoinjuries have mainly hampered the efficacious application of the technique. Resveratrol (RES) is a natural polyphenol compound with potent antioxidant, immunomodulatory and anti-inflammatory properties (Li, et al., 2023). Hara et al. showed that RES enhanced embryonic development and reduced the level of oxidative stress in bovine oocytes after cryopreservation (Hara et al., 2018). In addition, RES supplementation during mouse oocyte vitrification was discovered to protect mitochondria from cryopreservation damage (Iwata, 2021). Nevertheless, the application of RES was limited due to its low solubility, photostability and poor bioavailability (Bohara et al., 2022).

In recent years, a novel cryopreservation strategy, nano-cryopreservation that combines nanotechnology and cryogenic engineering has emerged to further improve the efficiency of oocyte vitrification. For example, Li et al. showed that 0.05% HA-NPs would reduce recrystallization during rewarming and increase the maturation as well as the survival rate of porcine oocytes after cryopreservation (Li et al., 2016). Yaa et al. found that superparamagnetic Fe_3_O_4_ could protect GV stage oocytes from cryoinjury and increase the expression levels of genes (OCT4, SOX2 and CDX2) related to cell pluripotency and differentiation (Abbasi et al., 2021). In addition, it was found that the addition of polylactic acid-coated melatonin to Etoposide (ETP) not only reduced DNA damage, increased ATP levels, maintained mitochondrial distribution, but also increased the maturation rate of oocytes after vitrification (Lee et al., 2023). Since the hydrophobic property mainly accounts for the unstable activity of RES, we hypothesized that the disadvantages of traditional antioxidant would be ameliorated by the use of polymeric nanocarriers.

Therefore, in the present study, RES was encapsulated with PLGA, and we discovered that PLGA was an efficient carrier delivery. Subsequent study confirmed the biosafety and validity of PLGA-RES in ovine oocyte maturation. Moreover, the effect of PLGA-RES on oocyte cryopreservation and the underlying mechanism was explored systematically. Our findings will provide new insights into deciphering the role of nanoparticles in stress responses and be definitely benefit for the overall improvement of oocyte cryopreservation technique.

## 2 Materials and methods

### 2.1 Materials

PLGA (Sigma-805726), TRITC (Sigma-87918), RES (Sigma-R5010), PVA (Sigma-P8136), Dichloromethane (Sigma-650463), PBS (Sigma-2272), TCM-199 (Gibco-C11150500BT), BME AA (Sigma-B6766), MEM AA (Sigma-M7145), Glutamine (Sigma-G8540), BSA (Sigma-A1933), DMSO (Sigma-D2650), FSH (Solarbio-F8470), LH (Solarbio-L8040), FBS (Gibco-16000), Gentamicin (Merck-G1264), NaHCO_3_ (Wako-191-01305), HEPES (Sigma-H4034), Sodium Pyruvate (Wako-199-03062), E_2_ (Sigma-E2758), DPBS (Sigma-D8662), Ethylene Glycol (EG) (Sigma-324558), Trehalose (Sigma-T0167), 6-DMAP (Sigma-D2629), H_2_O (Sigma-W1503).

### 2.2 Preparation of RES loaded PLGA nanoparticles and quality assessment

PLGA-RES was synthesized and embellished by water-in-oil-in-water solvent evaporation technique (Lee et al., 2023). Simply put, the PLGA and RES were dissolved in dichloromethane under continuous agitation to obtain a mixture. PVA with a mass concentration of 5% was added to the mixture and stirred for 1 h to remove dichloromethane. Centrifuge at 4,000 rpm for 5 min to remove large particle precipitation, centrifuge at 13,000 rpm for 5-10 min to remove the supernatant to obtain secondary precipitation, and wash with water to obtain secondary precipitation twice, and then gently stir the washed solid-liquid mixture in the steam pool at room temperature until complete evaporation of the organic phase is achieved. After centrifugation, the amount of RES in the supernatant is assayed by a spectrophotometer and these NPs were labeled with TRITC for tracing. Particle size and zeta potential of PLGA-RES were determined by dynamic light scattering (DLS) using NanoBrook 90 plus PALS (Brookhaven, US).

### 2.3 Drug release rate assay

5 mg PLGA-RES was dispersed in 4 mL deionized water, which was placed in a dialysis device (Dialysis membrane: 30 nm). Then, the dialysis device was immersed in 100 mL PBS solution (1 mM, pH 7.2 - 7.4) and mechanically stirred at room temperature. Finally, the dispersion was taken out at the specified times (0 h, 4 h, 24 h, 48 h, 192 h) for determining concentration of RES.

### 2.4 Cumulus oocyte complexes (COCs) collection and IVM

The ovine ovaries collected from slaughterhouse were kept in 37°C prewarmed normal saline and transported to the laboratory within 2 h. By cutting follicles with scalpels, obtained COCs were transferred to IVM medium (M199 medium supplemented with 2 mg/mL NaHCO_3_, 0.1 mg/mL gentamicin, 2 mg/mL HEPES, 0.2 mg/mL sodium pyruvate, 0.1% (v/v) FBS, 0.001% (v/v) FSH, 0.001% (v/v) LH, 0.001% (v/v) E_2_) with different drugs added in a humidified 5% CO_2_ incubator at 38.5°C for 24 h. The COCs were divided into three groups: control (untreated), RES (IVM medium supplemented with 0.5 μM RES), and PLGA-RES (IVM medium supplemented with PLGA-RES which can provide 0.5 μM RES for IVM medium) groups. The maturation rate is calculated as the ratio of oocytes with first polar body extrusion (PBE) to total oocytes.

### 2.5 Parthenogenesis activation and *in vitro* embryo culture (IVC)

Matured COCs were transferred into 0.024% (m/v) hyaluronidase for cumulus cells removal. Then MII oocytes with first polar body extrusion were selected and cultured in IVC medium (SOF medium supplemented with 2.5 mg/mL BSA, 2.5% (v/v) BME AA, 5% (v/v) MEM AA, 1 mg/mL Gln) supplemented with 7% (v/v) ethanol for 7 min in the dark. Then the oocytes were incubated in 2 mmol/L 6-DMAP for 4 h. After washing, the activated oocytes were cultured in humidified air and 5% CO_2_ at 38.5°C. Cleavage stage and blastocyst embryos were examined at 48 h and 192 h after activation, respectively.

### 2.6 Oocytes vitrification and thawing

MII oocytes matured in medium supplemented with PLGA-RES (Fresh) were then vitrified with PLGA-RES (Vit + PLGA) or without PLGA-RES (Vit). Vitrification of MII oocytes was carried out by the open pull pipette method. In brief, MII oocytes were placed in the equilibrium solution (DPBS medium containing 7.5% (v/v) dimethyl sulfoxide (DMSO), 7.5% (v/v) EG, and 20% (v/v) fetal bovine serum) for 3 min, then the oocytes were transferred to the vitrification solution (DPBS medium containing 0.5 mol/L trehalose, 15% (v/v) EG and 15% (v/v) DMSO) and loaded onto the carrier, which was subsequently plunged into liquid nitrogen. The total time period from vitrification medium exposure to liquid nitrogen immersion was less than 1 min.

For thawing, the carrier stored in liquid nitrogen was rapidly submerged into the prewarmed solution of 1.0 M trehalose for 1min at 37°C. Then the oocytes were sequentially transferred into 0.5 M trehalose, 0.25 M trehalose, 0.125 M trehalose, and basal solution (DPBS solution containing 20% fetal bovine serum) for 3 min, 3 min, 3 min, 5 min, respectively. Finally, the warmed oocytes were cultured in the IVM medium for further use.

### 2.7 Oocytes survival evaluation

MII oocytes were incubated in 0.1% w/v Trypan Blue (Merck, Germany) at room temperature for 5 min. After washing, the oocytes were observed under a microscope. Cells shown in blue are considered as dead cells, and the survival rate is the ratio of the number of surviving cells to the total number of cells.

### 2.8 Intracellular ROS and GSH level assay

The oocytes were incubated in 10 μmol/L 2′,7′-DCFH-DA or 10 μmol/L Cell Tracker Blue (Invitrogen, Carlsbad, CA, USA) at 38.5°C in 5% CO_2_ incubator for 20 min. After washing, the stained oocytes were placed on slides, observed and photographed under an immunofluorescence microscope (Carl Zeiss, City, Germany). Under the same staining conditions and photographic parameters, the region of interest (ROI) was selected using Image-Pro Plus 6.0 software, and the mean fluorescence intensity (MFI) per unit area within the ROI was statistically analyzed.

### 2.9 MMP detection

The MII oocytes were incubated in Rhodamine 123 staining working solution (1X) (Beyotime, China) at 38.5°C in 5% CO_2_ for 20 min. After washing, the stained oocytes were observed and photographed under the immunofluorescence microscope. Under the same staining conditions and photographic parameters, ROI was selected using Image-Pro Plus 6.0 software, and the MFI per unit area within the ROI was statistically analyzed.

### 2.10 Mitochondrial distribution assay

MII oocytes were placed in Mito-Tracker Green staining working solution (200 nM) (Beyotime, China) and incubated at 38.5°C, 5% CO_2_ for 20 min. After washing, the stained oocytes were observed under immunofluorescence microscope and photographed. Uniform green fluorescence was considered as normal mitochondrial distribution, whereas the hollows and aggregated fluorescence were indicated as abnormal mitochondrial distribution.

### 2.11 Intracellular calcium level detection

MII oocytes were placed in cytoplasmic calcium ion-specific probe Fluo-3 AM working solution (5 μmol/L) (Beyotime, China) and incubated at 38.5°C, 5% CO_2_ for 20 min. After washing, the stained oocytes were observed under an immunofluorescence microscope and photographed. Under the same staining conditions and photographic parameters, ROI was selected using Image-Pro Plus 6.0 software, and the MFI per unit area within the ROI was statistically analyzed.

### 2.12 RNA-seq analysis

Fresh, vitrified and PLGA-RES treated vitrified oocytes were collected and a set of three replicates were conducted in each group. After lysis and amplification, libraries were constructed. Selected cDNAs that met the quality requirements for concentration and fragment size were sequenced using SE50 on the BGISEQ platform. After cutting out sequencing aptamers and low-quality bases, quality control reads were aligned to ovine reference genome (ARS-UI_Ramb_v2.0) using HISAT version 2.2.1. After classification with Samtools version 1.12, gene reads were calculated with StringTie version 2.16. Differentially expressed genes (DEGs) was examined and multiple comparisons were performed using Student’s t-test and Benjamini & Hochberg (BH) method.

### 2.13 Immunofluorescence (IF) staining

Oocytes were fixed with 4% (w/v) paraformaldehyde (PFA) for 40 min at room temperature, followed by permeabilization with 0.5% Triton X-100 at room temperature for 1 h. After being blocked in 3% BSA for 1 h at room temperature, oocytes were incubated with different primary antibodies (anti-ARRB2, 1:500; anti-ULK3, 1:1000) overnight at 4°C. The oocytes were further incubated with an appropriate secondary antibody for 1 h at room temperature. Finally, fluorescent images were taken with immunofluorescence microscopy. Under the same staining conditions and photographic parameters, ROI was selected using Image-Pro Plus 6.0 software, and the MFI per unit area within the ROI was statistically analyzed.

### 2.14 Statistical analysis

Data are presented as mean ± SEM from at least three independent reduplicative experiments. Statistical analyses were performed using One-way ANOVA and Student’s t-test. ns: *p* > 0.05, *p < 0.05, and **p < 0.01.

## 3 Results

### 3.1 Characterization of PLGA-RES

**FIGURE 1:**
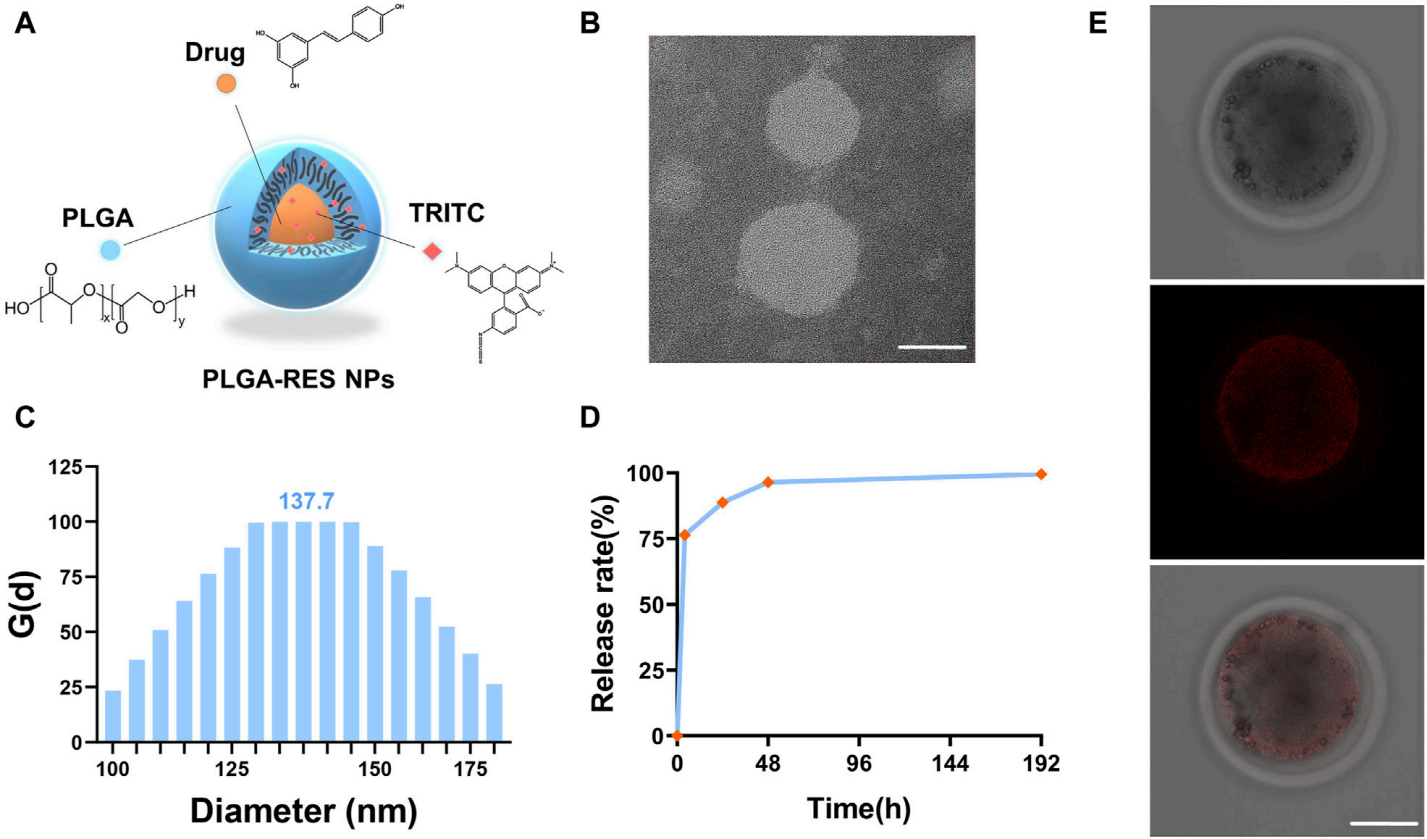
Characterization of PLGA-RES and their application to ovine oocytes. **(A)** A brief schematic illustration of synthesized PLGA-RES. **(B)** TEM image of PLGA-RES. Scale bar: 50 nm. **(C)** Size of NPs determined by DLS. **(D)** Cumulative release profiles of RES from PLGA-RES. **(E)** Confocal laser scanning microscopy images of NP-treated oocytes. PLGA-RES (red). Scale bar: 50 µm.

To trace the drug, biodegradable PLGA NPs were simultaneously loaded with RES and TRITC (Figure 1A). The biophysical properties of PLGA-RES nanoparticles, such as the morphology, diameter, and the surface charge, were determined. Transmission electron microscopy (TEM) images showed that the PLGA-RES presented a spherical shape and there was no aggregation between the prepared nanocarriers (Figure 1B). Dynamic light scattering (DLS) analysis showed that the average diameter of the particles was 137.7 nm (Figure 1C). By monitoring the zeta potential, the surface charge of the PLGA-RES was near neutral at 6.46 mV. In order to ensure its sustained release, we conducted *in vitro* dissolution tests, the release curve showed that RES was released rapidly in the first 48 h and smoothly discharged in the following 144 h, the release rate of RES reached 88.7% after 24 h incubation (Figure 1D).

### 3.2 The effect of PLGA-RES on oocytes survival, maturation and development

**FIGURE 2:**
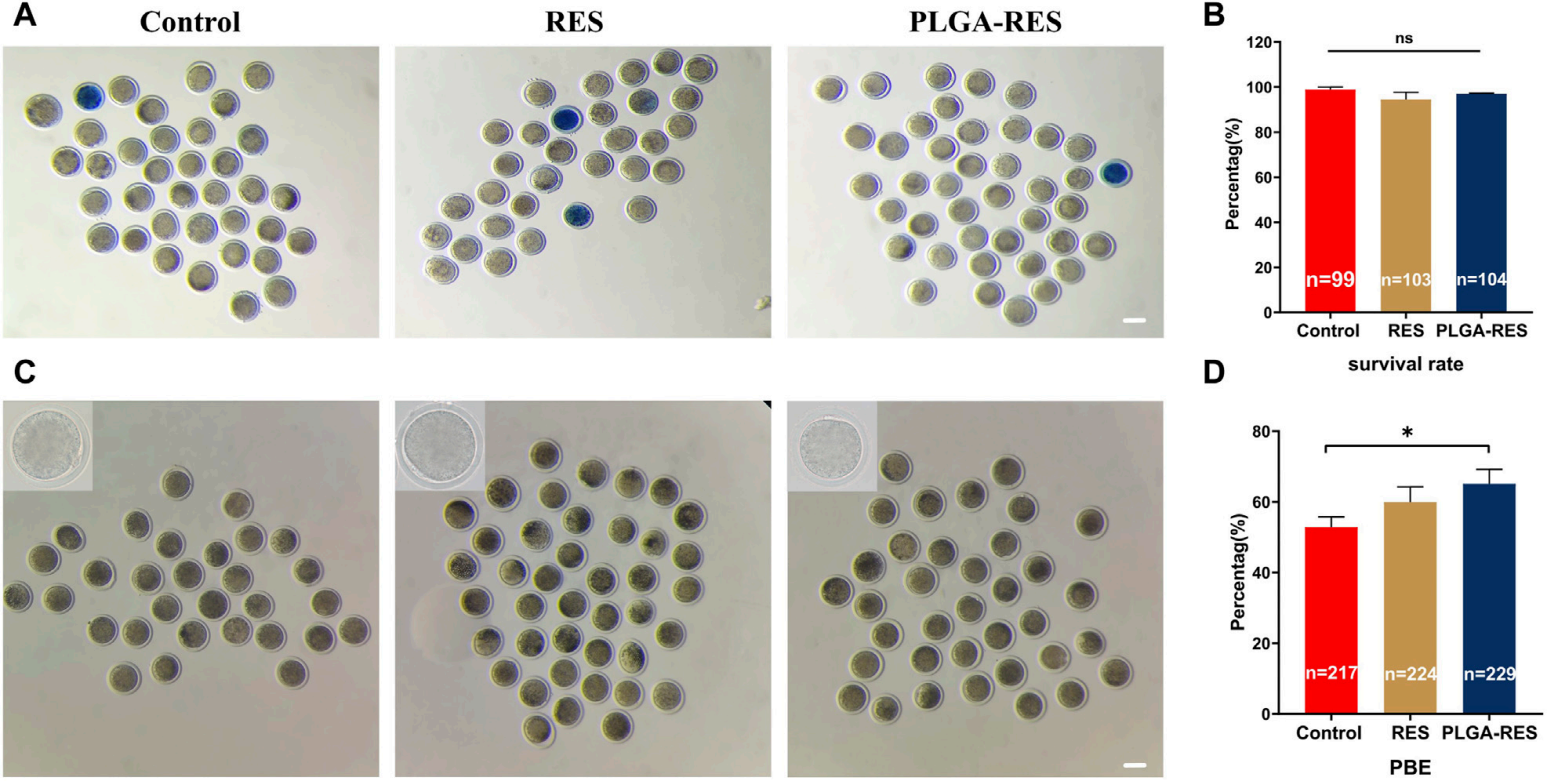
Effects of PLGA-RES on survival and maturation rate of ovine oocytes. **(A)** Representative images of survival oocytes. Scale bar = 100 μm. **(B)** Oocyte survival rate in the control, RES and PLGA-RES treatment groups. **(C)** Representative images of PBE. Scale bar = 100 μm. **(D)** PBE rate in the control, RES and PLGA-RES treatment groups. “n” represents the cell number used in this experiment. Data are presented as mean percentage (mean ± SEM) of at least three independent experiments. *p < 0.05, **p < 0.01, ns non significance.

**FIGURE 3:**
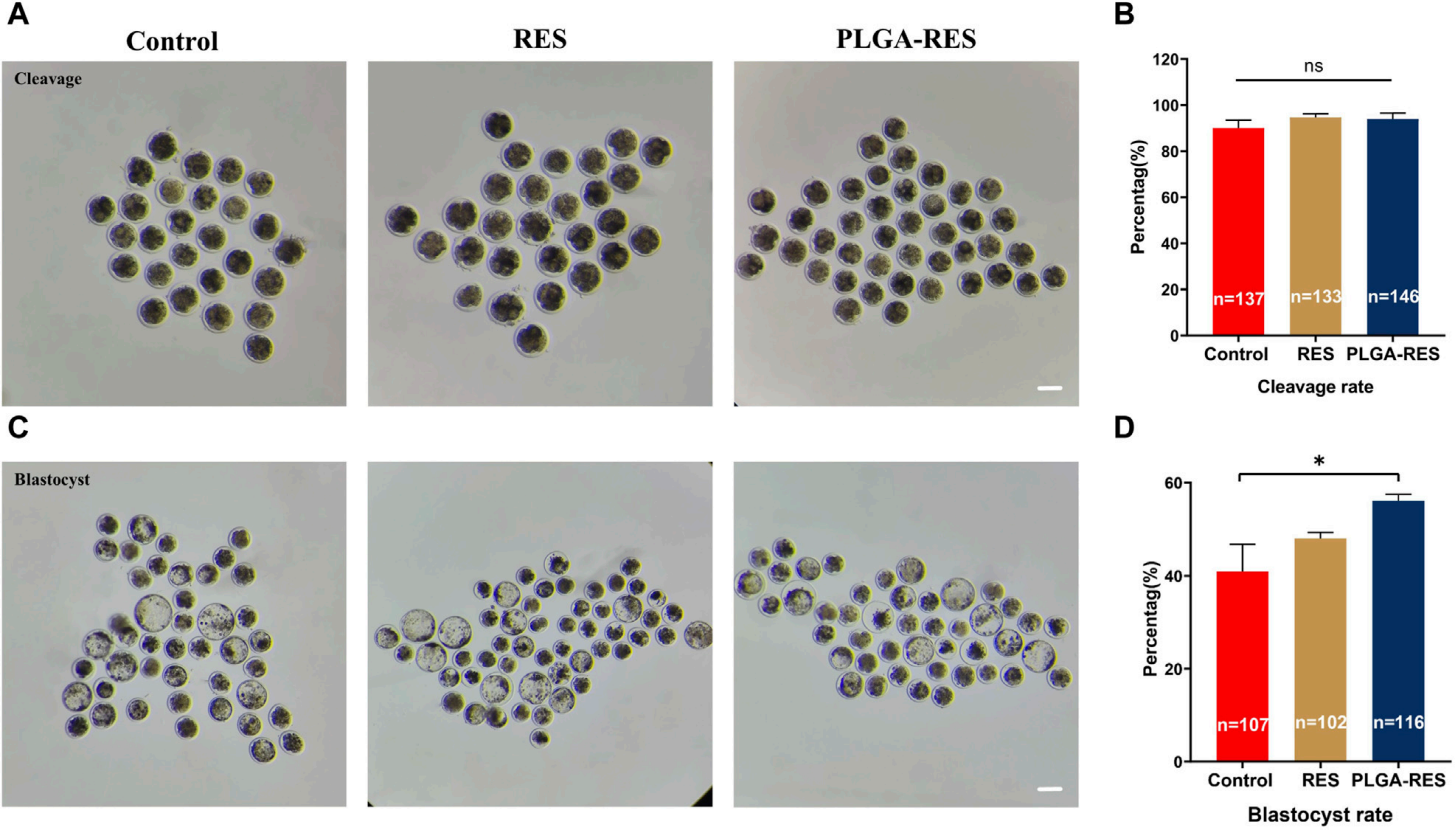
Effect of PLGA-RES on embryo development. **(A)** Representative images of cleavage embryos. Scale bar = 100 μm. **(B)** The cleavage rate in the control, RES and PLGA-RES treatment groups. **(C)** Representative images of blastocysts. **(D)** The blastocyst rate in the control, RES and PLGA-RES treatment groups. “n” represents the cell number used in this experiment. Data are presented as mean percentage (mean ± SEM) of at least three independent experiments. *p < 0.05, **p < 0.01, ns non significance.

To assess whether PLGA-RES can penetrate granulosa cells, zona pellucida (ZP), and cell membranes into the cytoplasm, GV stage oocytes were exposed to culture medium containing PLGA-RES for 24 h. Confocal fluorescence images showed that after 24 h of incubation, PLGA-RES could cross the ZP and cell membrane, and stay in the cytoplasm (Figure 1E). Next, the effects of PLGA-RES were evaluated after its penetrating into the cytoplasm. As shown in Figures 2A, B, the survival rate of PLGA-RES (97.08% ± 0.24% vs. 98.89% ± 1.11%, *p* > 0.05) and RES (94.49% ± 3.21% vs. 98.89% ± 1.11%, *p* > 0.05) supplementation group was not differed from that of the control group. The PBE rate of oocytes in PLGA-RES nanoparticles group was significantly higher than that of the control group (65.10% ± 4.11% vs. 52.85% ± 2.87%, *p* < 0.05), but there was no significant difference between the RES and control group (Figures 2C, D). Moreover, the effect of PLGA-RES on the developmental potential of embryos was explored. Our results showed that the addition of PLGA-RES group had no significant effect on the cleavage rate (94.20% ± 1.99% vs. 91.3% ± 2.94%, *p* > 0.05, Figures 3A, B), but significantly increased the blastocyst rate (56.13% ± 1.36% vs. 40.91% ± 5.85%, *p* < 0.05, Figures 3C, D).

### 3.3 PLGA-RES attenuated oxidative stress in IVM oocytes

**FIGURE 4:**
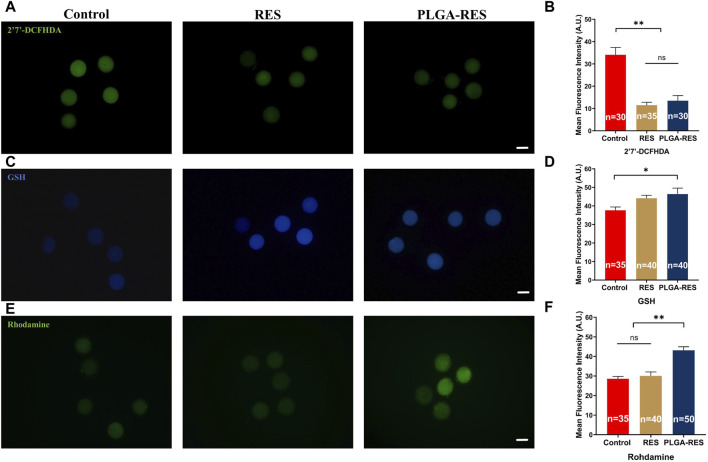
Effects of PLGA-RES on redox status of MII oocytes. **(A)** ROS signals of oocytes in the control, RES and PLGA-RES treatment groups. Scale bar = 100 μm. **(B)** The fluorescence intensity of ROS signals in different groups. **(C)** GSH signals of oocytes in different groups. Scale bar = 100 μm. **(D)** The fluorescence intensity of GSH signals in different groups. **(E)** MMP signals of oocytes in different groups. Scale bar = 100 μm. **(F)** The fluorescence intensity of MMP signals in different groups. “n” represents the cell number used in this experiment. Data are presented as mean percentage (mean ± SEM) of at least three independent experiments. *p < 0.05, **p < 0.01, ns non significance. A.U. represents Arbitrary Unit.

The oxidative stress indicators, including ROS, GSH and MMP levels were measured to examine the antioxidant capacity of PLGA-RES. ROS levels in oocytes from RES (11.49 ± 1.30 vs. 34.07 ± 3.30, *p* < 0.01) and PLGA-RES nanoparticle groups (13.49 ± 2.30 vs. 34.07 ± 3.30, *p* < 0.01) were significantly lower than that in the control group (Figures 4A, B). What’s more, as shown in Figures 4C, D, the GSH content in the PLGA-RES group were significantly higher than that of the control group (44.13 ± 1.57 vs. 37.62 ± 1.79, *p* < 0.01), and the MMP level in the PLGA-RES nanoparticles treatment group was much higher than that of the control (43.10 ± 1.81 vs. 28.52 ± 1.25, *p* < 0.01) and RES (43.10 ± 1.81 vs. 30.08 ± 1.98, *p* < 0.01) group (Figures 4E, F).

### 3.4 PLGA-RES improved oocytes quality after vitrification

**FIGURE 5:**
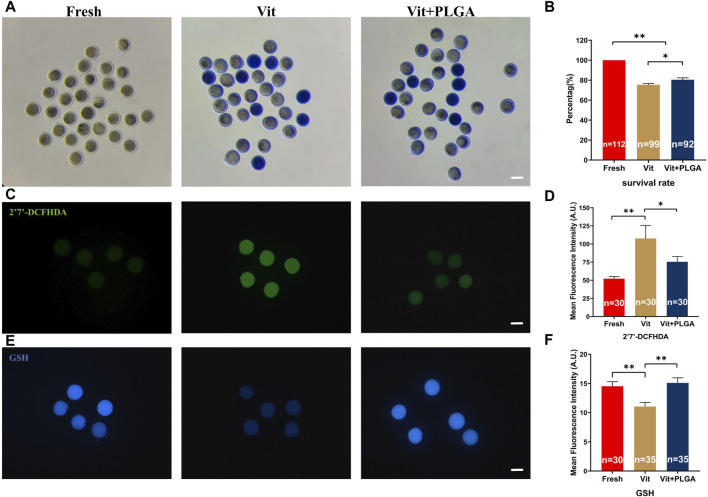
Effects of PLGA-RES on survival rate and redox status of vitrified-thawed MII oocytes. **(A)** Representative images of survival oocytes after thawing. Scale bar = 100 μm. **(B)** Survival rate in the fresh, vitrified, and PLGA-RES-treated vitrified oocytes. **(C)** Representative images of ROS signals after thawing. Scale bar = 100 μm. **(D)** The fluorescence intensity of ROS signals in different groups. **(E)** Representative images of GSH signals after thawing. Scale bar = 100 μm. **(F)** The fluorescence intensity of GSH signals in different groups. “n” represents the cell number used in this experiment. Data are presented as mean percentage (mean ± SEM) of at least three independent experiments. *p < 0.05, **p < 0.01, ns non significance. A.U. represents Arbitrary Unit.

To investigate the effect of PLGA-RES on oocytes quality during vitrification, survival rate of oocytes was determined. Our results showed that the survival rates of Vit (75.37% ± 1.3% vs. 100%, *p* < 0.01) and Vit + PLGA (80.42% ± 1.97% vs. 100%, *p* < 0.01) groups were significantly lower than that of the Fresh group, and the Vit + PLGA group survival rates were significantly higher than Vit group (80.42% ± 1.97% vs. 75.37% ± 1.3%, *p* < 0.05) (Figures 5A, B). Furthermore, the effect of PLGA-RES on oxidative stress of vitrified oocytes was investigated. The ROS level in the Vit group was dramatically higher than that in the control group (107.61 ± 17.96 vs. 52.11 ± 2.95, *p* < 0.01), while in the Vit + PLGA group, the ROS level was significantly lower than that in the Vit group (75.41 ± 7.23 vs. 52.11 ± 2.95, *p* < 0.05) (Figures 5C, D). Moreover, the level of GSH in the Vit group was highly significantly lower than that in the control group (11.04 ± 0.71 vs. 14.51 ± 0.78, *p* < 0.01), whereas, the GSH level in the Vit + PLGA group was significantly higher than that in the Vit group (15.09 ± 0.86 vs. 14.51 ± 0.78, *p* < 0.01) (Figures 5E, F).

### 3.5 PLGA-RES restored mitochondrial function in vitrified oocytes

**FIGURE 6:**
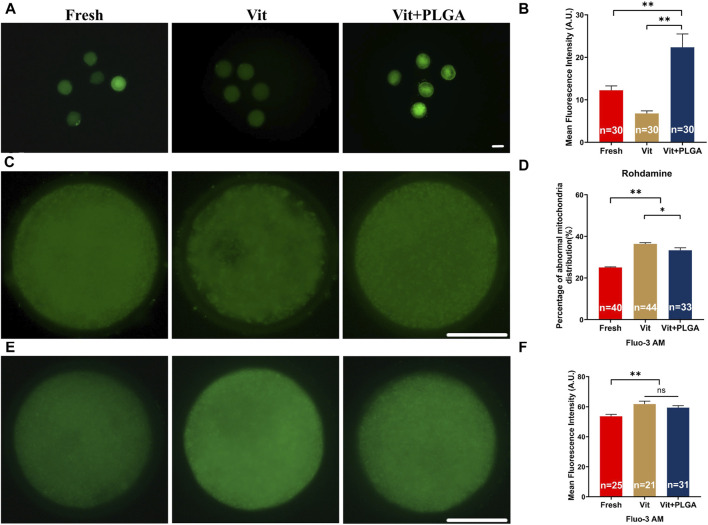
Effects of PLGA-RES on mitochondria function and calcium level in vitrified oocytes. **(A)** Representative images of MMP signals. Scale bar = 100 μm. **(B)** The fluorescence intensity of MMP signals in the fresh, vitrified, and PLGA-RES-treated vitrified oocytes. **(C)** Representative images of mitochondrial distribution in different groups. Scale bar = 50 μm. **(D)** The ratio of abnormal mitochondrial distribution in different groups. **(E)** Representative images of cytoplas-mic calcium in different groups. Scale bar = 50 μm. **(F)** The fluorescence intensity of Fluo-3 AM signals in different groups. “n” represents the cell number used in this experiment. Data are presented as mean percentage (mean ± SEM) of at least three independent experiments. *p < 0.05, **p < 0.01, ns non significance. A.U. represents Arbitrary Unit.

Because of pivotal role of mitochondria in redox homeostasis regulation, MMP level, mitochondrial distribution and intracellular Ca^2+^ levels that reflects its function were measured after PLGA-RES treatment of vitrified oocytes. The MMP was significantly increased in the Vit + PLGA group when compared with the Fresh (22.56 ± 3.15 vs. 12.24 ± 1.02, *p* < 0.01) and Vit (22.56 ± 3.15 vs. 6.79 ± 0.60, *p* < 0.01) groups (Figures 6A, B). Mitochondrial abnormality distribution rate in the Vit group was significantly higher than that in the Fresh group (33.33% ± 1.15% vs. 36.36% ± 0.58%, *p* < 0.05), whereas, the mitochondrial abnormality distribution rate was markedly declined in the Vit + PLGA group (25.00% ± 0.29% vs. 33.33% ± 1.15%, *p* < 0.01). Furthermore, the mitochondrial abnormality distribution rate was significantly reduced in Vit + PLGA group compared with that of the Vit group (25.00% ± 0.29% vs. 33.33% ± 1.15%, *p* < 0.01) (Figures 6C, D). The intracellular Ca^2+^ content was significantly higher in the Vit (61.76% ± 1.99% vs. 53.66% ± 1.32%, *p* < 0.01) and Vit + PLGA group (59.34% ± 1.32% vs. 53.66% ± 1.32%, *p* < 0.01), when compared with that of the Fresh group (Figures 6E, F).

### 3.6 DEGs induced by PLGA-RES were identified by transcriptome analysis

**FIGURE 7:**
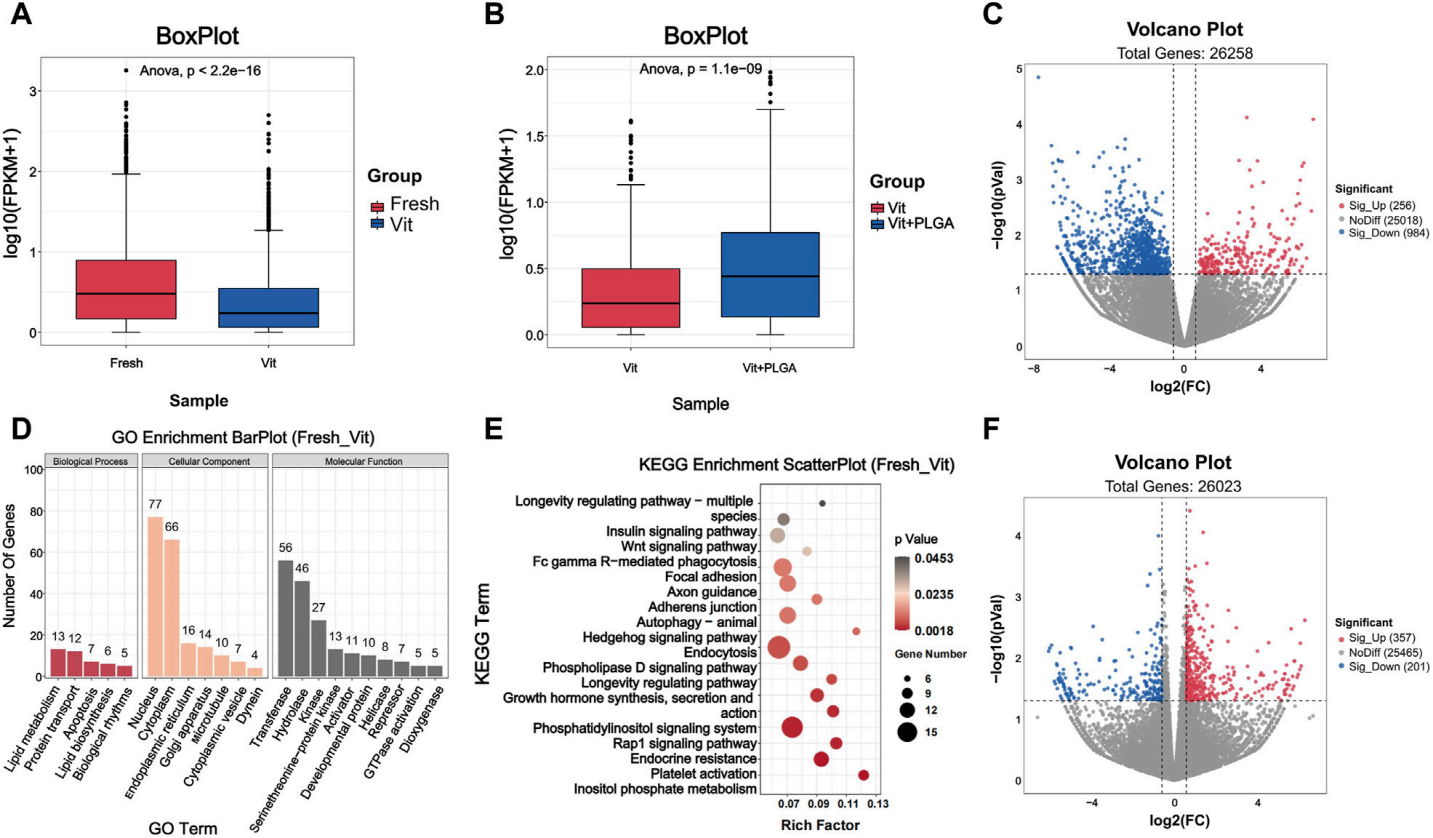
Quality assessment of RNA-seq data and analysis of DEGs. **(A)** Boxplot of RNA-seq differences between Fresh and Vit groups. **(B)** Boxplot of RNA-seq differences between Vit and Vit + PLGA groups. **(C)** Volcano plot of genes information for RNA-seq compared with Fresh group and Vit group. **(D)** Bar plot of GO classification of DEGs compared with Fresh and Vit group. **(E)** Scatter plot of KEGG enrichment of DEGs compared with Fresh and Vit group. **(F)** Volcano plot of genes information for RNA-seq compared with Vit group and Vit + PLGA group.

To further investigate the potential molecular mechanisms underlying the protective role of PLGA-RES during oocytes vitrification, we compared Fresh with Vit groups, and Vit with Vit + PLGA groups by RNA-seq analysis. To verify the statistical repeatability of the three groups, the expression level of each gene was calculated using the number of exon model Fragments Per Kilobase of exon model per Million mapped fragments (FPKM). Statistical analysis of FPKM showed that the distribution ranges of genes differed significantly among different subgroups (Figures 7A, B).

The functional classification and enrichment analysis of DEGs in Fresh with Vit groups, and Vit with Vit + PLGA groups were performed. In order to screen gene regulatory pathways of oocytes after vitrification, Fresh and Vit groups comparisons were performed. The results showed that there were 1240 DEGs in the Vit group compared to the Fresh group, of which 256 genes were upregulated and 984 genes were downregulated (Figure 7C). GO analysis showed that DEGs were mainly enriched in nucleus, cytoplasm, apoptosis, transferase and hydrolase (Figure 7D). KEGG analysis showed that DEGs were mainly enriched in signaling pathways such as endocytosis, Rap1 signaling pathway, autophagy, and Wnt signaling pathway in the Vit group compared to the Fresh group (Figure 7E).

**FIGURE 8:**
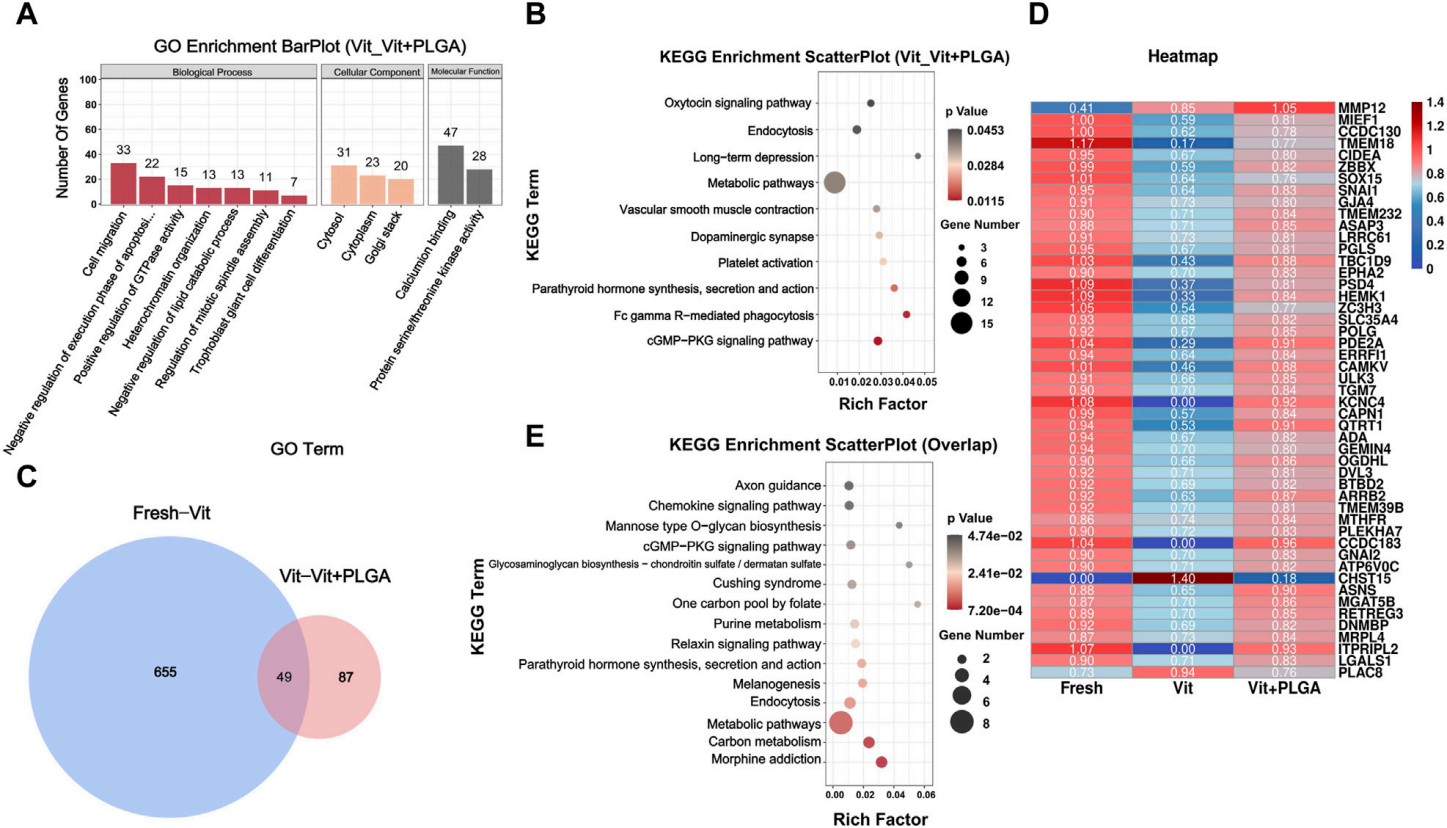
GO classification and KEGG enrichment analysis of DEGs from RNA-seq. **(A)** Bar plot of GO classification of DEGs from Vit - Vit + PLGA group. **(B)** Scatter plot of KEGG enrichment of DEGs from Vit - Vit + PLGA group. **(C)** Venn diagram of overlapped genes from Fresh - Vit, Vit - Vit + PLGA. **(D)** Expression comparison of overlapping genes from Fresh - Vit, Vit - Vit + PLGA. **(E)** Scatter plot of KEGG enrichment of overlap genes from Fresh - Vit, Vit - Vit + PLGA.

In order to screen gene regulatory pathways of vitrified oocytes after PLGA-RES treatment, Vit and Vit + PLGA groups comparisons were performed. There were 558 DEGs in the Vit + PLGA group compared to the Vit group, of which 357 genes were upregulated and 201 genes were downregulated in the Vit + PLGA group (Figure 7F). DEGs between Vit + PLGA and Vit groups were mainly enriched in cell migration, negative regulation of execution phase of apoptosis, Golgi stack and calcium ion binding and protein serine/threonine kinase activity (Figure 8A). Compared with Vit group, DEGs in Vit + PLGA group were mainly enriched in endocytosis, Fc gamma R-mediated phagocytosis, metabolic pathway and cGMP-PKG (Figure 8B).

### 3.7 PLGA-RES mainly involved in endocytosis and PI3K/AKT/mTOR pathway regulation

In order to screen out the core genes in the three groups, determine their functions and common core regulatory networks after different treatment conditions, the DEGs of Fresh with Vit groups, and Vit with Vit + PLGA groups were overlapped. 49 targets were obtained by crossover analysis of DEGs (Figures 8C, D). In addition, the 49 genes enriched signaling pathways were endocytosis, chemokine signaling pathway, and cGMP-PKG signaling pathway (Figure 8E).

**FIGURE 9:**
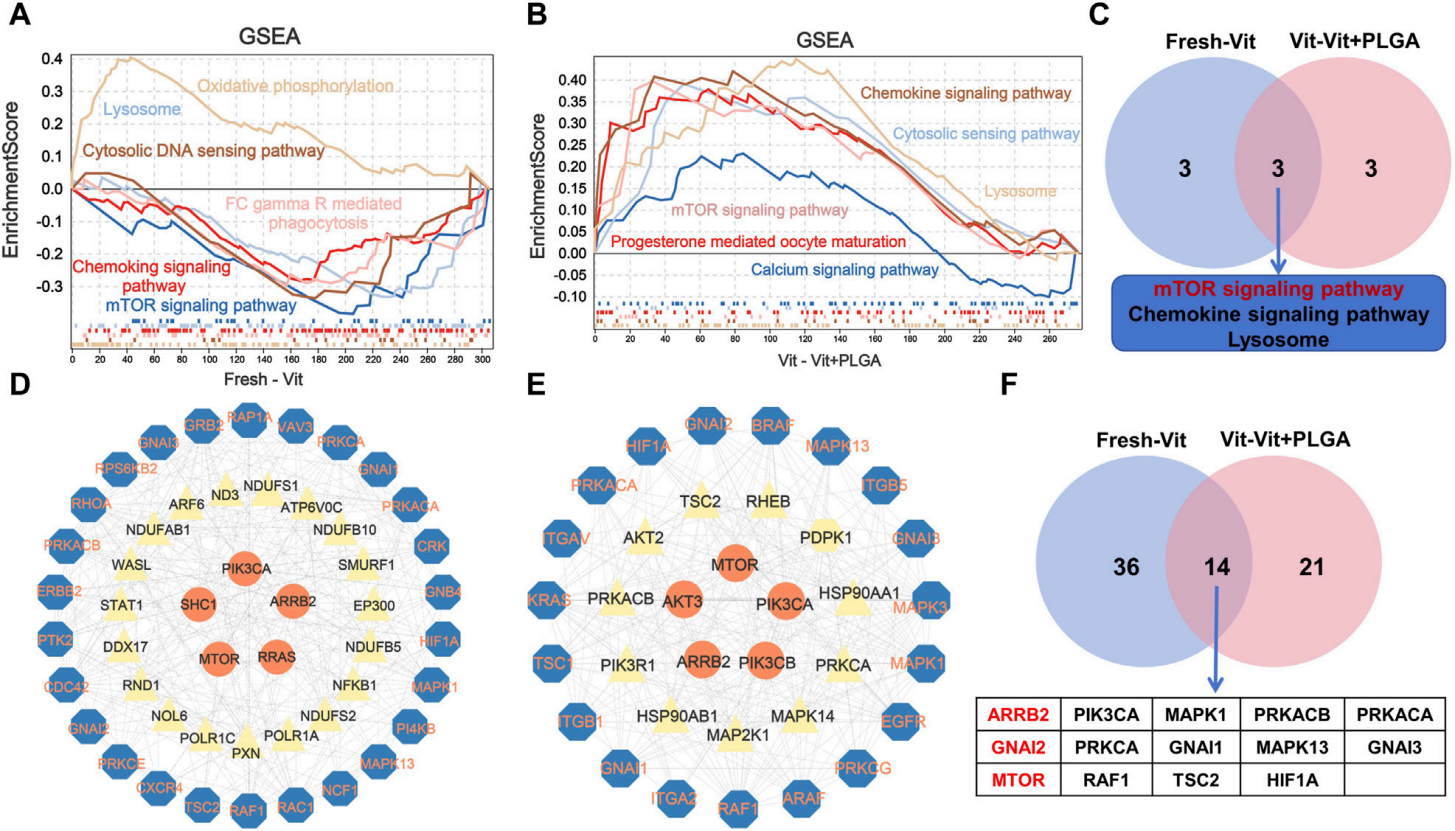
Enrichment analysis of GSEA based on the quantitative genes obtained from RNA-seq. **(A)** The GSEA analysis of quantitative genes from Fresh - Vit groups. **(B)** The GESA analysis of quantitative genes from Vit - Vit + PLGA groups. **(C)** The VENN diagram of the overlapping pathways from Fresh - Vit and Vit - Vit + PLGA groups in GSEA. **(D)** Interaction networks constructed by the genes expressed in Fresh - Vit groups. **(E)** Interaction networks constructed by the genes expressed in Vit - Vit + PLGA groups. **(F)** The VENN diagram of the overlapping genes from Fresh - Vit and Vit - Vit + PLGA groups in PPI.

To explore the overall trend of quantitative genes in the transcriptome, all quantitative genes were subjected for GSEA analysis, and the results showed that the pathways enriched by Fresh - Vit were mTOR signaling pathway, lysosome, cell membrane DNA sensing pathway, chemokine signaling pathway, oxidative phosphorylation, and Fc gamma R-mediated phagocytosis (Figure 9A), and the pathways enriched for Vit - Vit + PLGA were mTOR signaling pathway, lysosome, cell membrane DNA sensing pathway, calcium signaling pathway, ECM receptor interactions, and progesterone mediated oocyte maturation (Figure 9B). We compared the two sets of pathways screened in GSEA and found that the overlapped pathways were mTOR signaling pathway, lysosome and chemokine signaling pathway (Figure 9C).

We then performed protein network interactions and core gene screening analysis on the genes screened by GO, KEGG and GSEA. A total of 50 core genes in Fresh - Vit were screened, including *MTOR*, *ARRB2*, *GNAI2*, *GNAI1*, *RAC1*, *RAF1*, etc (Figure 9D), A total of 35 core genes in Vit - Vit + PLGA were screened, including *MTOR*, *GNAI2*, *AKT3*, *PIK3CA*, *AKT2*, *ARRB2*, *AKT3*, *PIK3CA,* etc (Figure 9E). There were 14 core genes that overlap Fresh - Vit and Vit - Vit + PLGA, including *ARRB2*, *GNAI2*, *MTOR*, *PIK3CA*, *PRKCA*, *RAF1*, *MAPK1*, *GNAI1*, *TSC2*, *PRKACB*, *MAPK13*, *HIF1A*, *PRKACA* and *GNAI3* (Figure 9F).

### 3.8 Declined expression levels of ARRB2 and ULK3 in vitrified oocytes were rescued by PLGA-RES treatment

**FIGURE 10:**
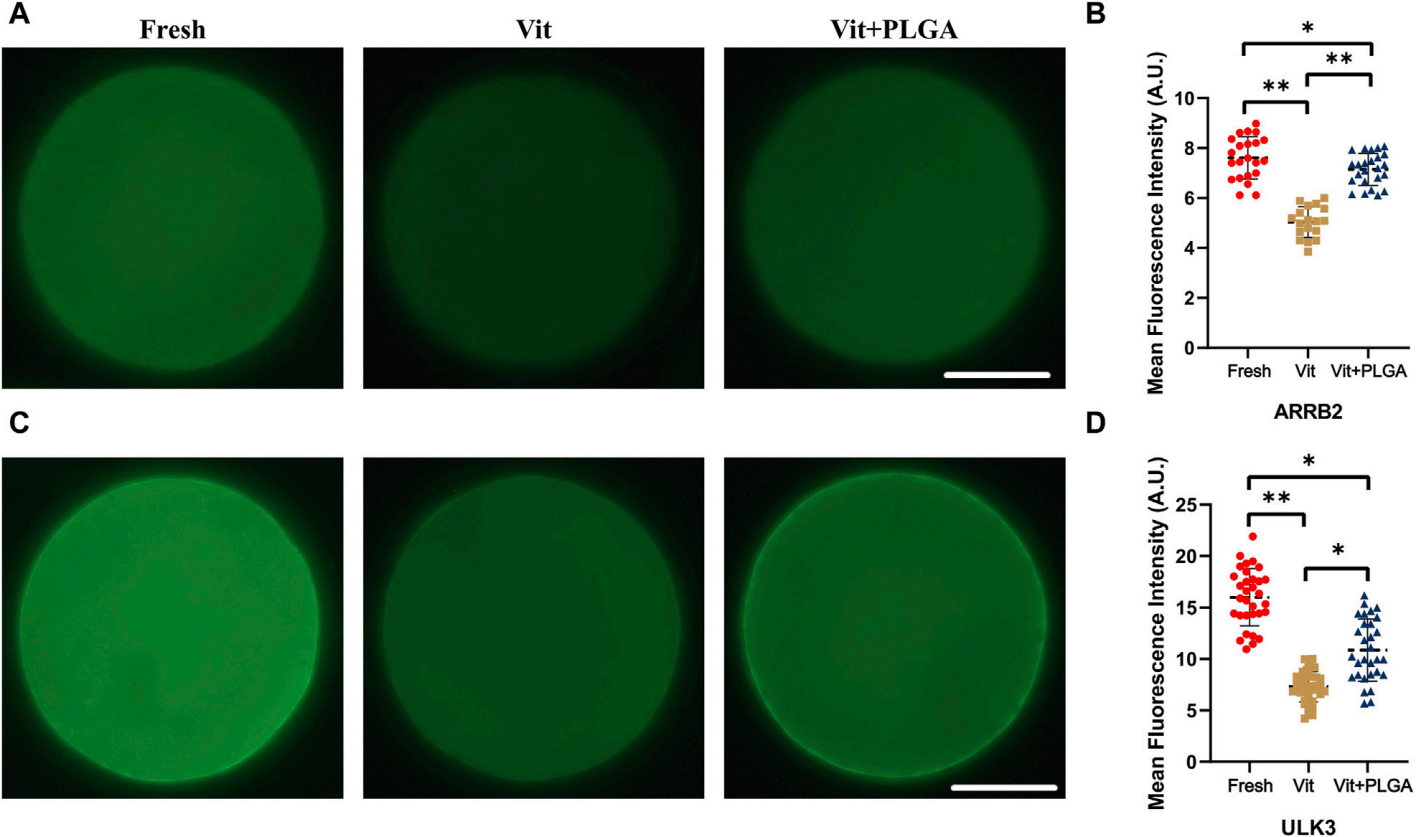
Effect of PLGA-RES on the expression of ARRB2 and ULK3. **(A)** Representative images of ARRB2 signals in fresh, vitrified, and PLGA-RES treated vitrified oocytes. Scale bar = 50 μm. **(B)** The fluorescence intensity analysis of ARRB2 signals in different groups. **(C)** Representative images of ULK3 signals in different groups. Scale bar = 50 μm. **(D)** The fluorescence intensity analysis of ULK3 signals in different groups. A.U. represents Arbitrary Unit.

The expression levels of two key genes from transcriptome integration analysis were verified. The results showed that the expression of *ARRB2* in vitrified oocytes was significantly lower than that in the control group (5.03 ± 0.15 vs. 7.63 ± 0.2, *p* < 0.01), and the declined ARRB2 level was markedly reversed by PLGA-RES treatment (7.15 ± 0.13 vs. 5.03 ± 0.15, *p* < 0.05). However, the ARRB2 level in the Vit + PLGA group was still lower than that of the control group (7.15 ± 0.13 vs. 7.63 ± 0.2, *p* < 0.05) (Figures 10A, B). In addition, we found that the expression of *ULK3* was dramatically reduced in vitrified oocytes (7.30 ± 0.24 vs. 15.74 ± 0.54, *p* < 0.01), and the declined ULK3 level was markedly reversed by PLGA-RES treatment (10.87 ± 0.55 vs. 7.30 ± 0.24, *p* < 0.01). However, the ULK3 level in the Vit + PLGA group was still lower than that of the control group (10.87 ± 0.55 vs. 15.74 ± 0.54, *p* < 0.05, Figures 10C, D).

## 4 Discussion

The egg coat, known as ZP is one of the most distinguished features in oocytes. Composed of glycoproteins, ZP is the outer layer of the oocyte and preimplantation embryo, and is a unique barrier that effectively prevents the physical invasion of foreign substances (Moros-Nicolas et al., 2021). We therefore firstly investigated whether PLGA-RES would penetrate ZP. Our results showed that PLGA-RES could enter the oocyte through the granulosa cells and ZP, indicating that PLGA-RES can be absorbed normally by ovine oocytes. PLGA has been reported to degrade slowly in the physiological environment or at the tumour location, breaking down into lactic acid (LA) and glycolic acid (GA), both of which are physiological metabolites of the citric acid cycle (Su, et al., 2021). To evaluate the effect of the newly constructed NPs on oocytes, the survival and maturation rates of oocytes treated with PLGA-RES were investigated. The results showed that the survival rate of oocytes in the PLGA-RES group was not significantly different from that of the control or the RES treated counterparts, however, the PBE rate was significantly increased by the addition of PLGA-RES, indicating that the PLGA-RES not only had low biotoxicity but also was beneficial to oocyte maturation *in vitro*. Our data was similar with the results of Kim et al. who found that PLGA would not compromise the quality of mouse preimplantation embryos (Kim, et al., 2018). It is noteworthy that PLGA-RES can enhance oocyte development to a greater extent than conventional RES administration. It is known that the controlled release rate was one of the advantages of nanomaterials. It has been shown that the addition of PLGA during bovine oocyte maturation was detected up to the blastocyst stage (Gonçalves, et al., 2021). Thus, we believe that it is the slow-release property of the nanoparticle that allows for a sustained release of the loaded drug in our experiment, which substantially prolongs the half-life of the drug. Our research indicated that the drug release rate of the PLGA-RES reached 88.7% during the first 24 h, which was much higher than that of the reported 80% (Wan et al., 2018), 78% (Bhatt et al., 2020), 55% (Aldawsari et al., 2020), and 50% (Kumar et al., 2015) in the previous studies.

ROS are produced by a variety of cellular metabolic activities, particularly as a byproduct of mitochondrial respiration mediated ATP production, however, conditions in which ROS is over-accumulated or highly reduced can disrupt REDOX homeostasis, lead to oxidative stress, and impair development in oocytes through multiple mechanisms (Hardy et al., 2021). GSH acts as a natural antioxidant inside cells to inhibit oxidative stress (Adeoye et al., 2018). Many studies have reported that ROS, GSH and MMP were indicators of oxidative stress on oocytes (Zhuan et al., 2022; Bai et al., 2023; Sun et al., 2023). To further evaluate the effect of PLGA-RES on oxidative stress, ROS, GSH and MMP levels were measured, and the results indicated that PLGA-RES dramatically reduce oxidative status in in vitro matured oocytes. Notably, we found that PLGA-RES was superior to RES in ameliorating mitochondrial function. The factors contributed to the better therapeutic efficacy of PLGA nano-encapsulation are as follows: i) nano-encapsulation makes hydrophobic drugs hydrophilic and thus more effective in entering the oocyte; ii) nano-encapsulated drugs have a slow-release effect, which prolongs the half-life of the drug; and iii) the metabolites of PLGA, such as lactic and acetic acids, are biodegradable compounds. Previous study reported that lactic acid is a signaling molecule in the PKA signaling pathway of bovine granulosa cells (Baufeld and Vanselow, 2022), and it participated in cellular metabolism and redox process regulation (Brooks et al., 2023). Therefore, the released lactic acid from PLGA NPs degradation would also exert antioxidant function and potentially protect oocytes against oxidative stress. It can be speculated that the multiple properties of PLGA-RES promote maturation by inhibiting oxidative stress in ovine oocytes.

The application of nanotechnology provides new alternative for further improving the efficiency of vitrification technique. At present, the role of PLGA-RES played in oocyte cryopreservation has not been identified. In this study, we applied PLGA-RES to the vitrification and thawing medium, and investigated its effect on oocytes quality. We found that the survival rate was markedly increased when oocytes were treated with NPs during vitrification, which was in similar with the previous finding that melatonin encapsulated in PLGA was efficacious in mouse GV oocytes vitrification (Lee et al., 2023). Moreover, the effect of PLGA-RES on oxidative stress was evaluated, and the results showed that the ROS level was reduced while GSH content was increased in oocytes treated with PLGA-RES during vitrification. Since decreased oocyte quality has been reported to be associated with elevated ROS and decreased MMP (Kirillova et al., 2021), the present finding indicated that PLGA-RES maintained the developmental potential under vitrification stress. Besides, our results demonstrated that PLGA-RES still exhibited antioxidant properties under cryogenic treatment. Zhang et al. also concluded that the release properties of PLGA NPs was not rendered by the low temperature (Zhang et al., 2019). As central organelles for metabolism and ROS production, mitochondria supply large amounts of ATP during meiosis and exhibit organelle rearrangements during nuclear and cytoplasmic maturation through mtDNA replication and mitochondrial dynamics (Udagawa and Ishihara, 2020; Zou et al., 2021). It has been shown that vitrification can significantly affect the function and distribution of mitochondria in oocytes (Lei et al., 2014). In the present study, PLGA-RES significantly improved the abnormal distribution of mitochondria induced by vitrification. This indicated that PLGA-RES enhanced mitochondrial recovery in oocytes from damage caused by cryopreservation, which was similar to the results of Iwata’s study that RES can improve mitochondrial function in oocytes after cryopreservation. (Iwata, 2021). Cryoprotectant agent, specifically DMSO, has been shown to trigger endoplasmic reticulum Ca^2+^ release, resulting in abnormally high cytoplasmic and mitochondrial Ca^2+^ levels in bovine oocyte vitrification (Gualtieri et al., 2021). In the present study, sheep oocytes showed abnormal elevation of intracellular Ca^2+^ levels after vitrification, but intracellular Ca^2+^ levels did not change after treatment with PLGA-RES. Although PLGA-RES restored mitochondrial function to a certain extent, a portion of vitrification-induced Ca^2+^ could not flow into the medium in time due to ZP hardening caused by vitrification (Wiesak et al., 2017; Lan et al., 2022), leading to Ca^2+^ overload in the cytoplasm. In general, PLGA-RES had positive effects on the cryopreservation of MII oocytes.

Finally, transcriptomics sequencing was performed to investigate the mechanism by which PLGA-RES improve the quality of vitrified oocytes. And the results showed that the significantly enriched pathways were the Endocytosis pathway, suggesting that PLGA-RES are fully internalized by endocytosis like most other NPs. Previous study indicated that polymer-based NPs mostly enter the cell via Clathrin-mediated endocytosis based on binding of β-arrestin and G protein-coupled receptors (GPCRs) (Zhang et al., 2020; Janetzko et al., 2022; Szewczyk-Roszczenko et al., 2023; Wess et al., 2023). In the present study, we found that the expression of ARRB2 was restored in vitrified oocytes after PLGA-RES NPs treatment. ARRB2, also known as β-arrestin 2, is the member of β-arrestin subfamily, and is mainly involved in post-endocytosis transport through lysosomes (Zhang et al., 2008). And ARRB2 is an important protein that presents extracellular signals and promotes endocytosis, and the change of its expression will affect the degree of endocytosis (Urs et al., 2005; Abrisqueta et al., 2013; Tian et al., 2014; Zhang et al., 2020; Asher et al., 2022). Many studies have shown that endocytosis pathway with ARRB2 protein as the core plays an important role in drug uptake (Porter-Stransky and Weinshenker, 2017; Li et al., 2021). Our findings suggested that the elevated ARRB2 level would potentiate endocytosis process of PLGA-RES, which increased uptake drugs and extended release of drugs.

**FIGURE 11:**
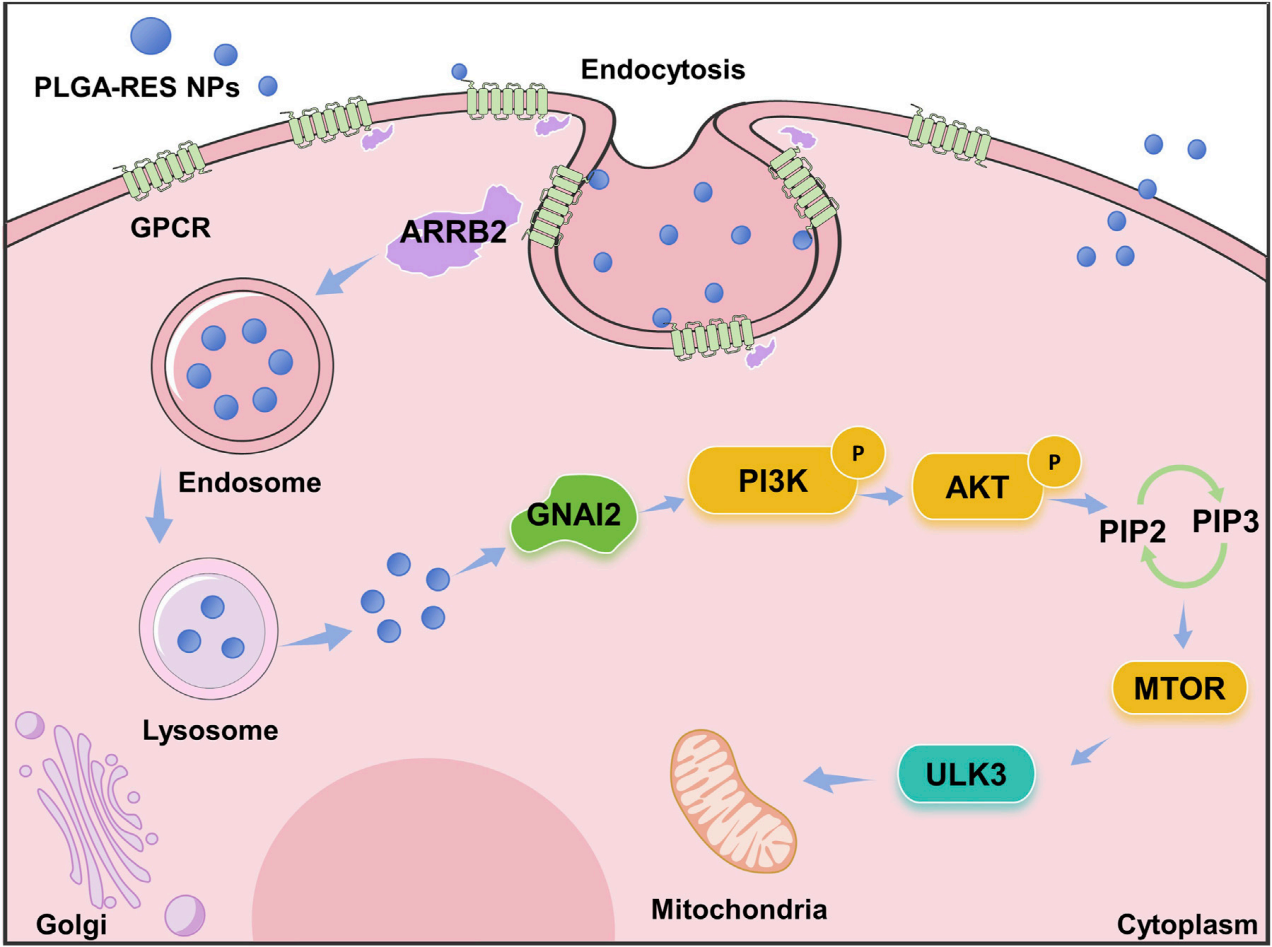
Schematic representation of the proposed mechanism underlying the protective role of PLGA-RES in oocytes vitrification.

Previous study indicated that large amounts of ROS generated during vitrification would result in oxidative stress which reduced oocytes developmental potential (Shirzeyli et al., 2021). Therefore, it was not surprisingly that genes involved in oxidative stress-related pathway, the PI3K/AKT/mTOR pathway, was differentially expressed in our study. PI3K/AKT/mTOR pathway participated in autophagy which remove oxidized/damaged proteins and bulky ROS-generating organelles for restricting the production and devastating effect of ROS, when ROS levels are abnormally elevated (Dong et al., 2022; Zhou et al., 2022; Glaviano et al., 2023). Importantly, the serine/threonine protein kinase ULK3 on the PI3K/AKT/mTOR pathway is an upstream regulator of autophagy under the regulation of mTORC1, which positively regulates autophagy (Young et al., 2009; Braden and Neufeld, 2016; Cayo et al., 2021). As the bottom of the mTOR pathway, it directly regulates initiation of autophagy though involving in membrane targeting and curvature (Florey et al., 2011; Hill et al., 2019). In this study, we found that the PI3K/AKT pathway was activated after vitrification, and *ULK3*, a gene in the mTOR pathway that induces autophagy, was downregulated, thereby inhibiting autophagy. Autophagy is widely considered to be a survival or response mechanism triggered by various environmental and cellular stressors. And mTOR signaling pathway was participated in autophagy. It has been shown that autophagy plays a protective role in oocytes during vitrification and IVM, and PI3K/Akt/mTOR pathway is involved in regulating the autophagic activity of oocytes during vitrification and IVM, which is similar to our findings (Zhang et al., 2020). In addition, our result was also similar with the previous study that PLGA-melatonin was more effective in reducing DNA and mitochondrial damages compared with the free drug treatment due to the controlled release of melatonin (Lee et al., 2023). Last but not least, as indicated in Figure 11, we speculate that PLGA-RES are incorporated into oocytes through endocytosis, then the released NPs maintain mitochondrial function by elevating *ULK3*, a down-stream target of PI3K/AKT/mTOR pathway.

## 5 Conclusion

In summary, biocomposite nanoparticles PLGA-RES was found to protect oocytes against vitrification stimuli. For the first time, we found that the PLGA-RES improved survival and blastocyst rate during oocytes development and cryopreservation, which provide a comprehensive perspective on the effective application of novel composite nanoparticles and contribute to decipher the mechanism underlying the protective role of NPs. And our results demonstrate that PLGA-RES is superior in protecting vitrified oocytes from cryoinjuries via endocytosis and PI3K/AKT/mTOR pathway regulation. The PLGA-RES is significantly more effective than resveratrol alone reported by others. The direction of future work will be to further explore the mechanism of *ULK3* gene’s influence on mitochondrial function through the control of autophagy.

## Data Availability

The data presented in the study are deposited in the National Genomics Data Center repository, accession number PRJCA023884.
